# Radiolytic Reduction Characteristics of Artificial Oligodeoxynucleotides Possessing 2-Oxoalkyl Group or Disulfide Bonds

**DOI:** 10.4061/2011/816207

**Published:** 2011-08-08

**Authors:** Kazuhito Tanabe, Takeo Ito, Sei-ichi Nishimoto

**Affiliations:** Department of Energy and Hydrocarbon Chemistry, Graduate School of Engineering, Kyoto University, Katsura Campus, Kyoto 615-8510, Japan

## Abstract

A number of advances have been made in the development of modified oligodeoxynucleotides (ODNs), and chemical or physical properties of which are controlled by external stimuli. These intelligent ODNs are promising for the next generation of gene diagnostics and therapy. This paper focuses on the molecular design of artificial ODNs that are activated by X-irradiation and their applications to regulation of hybridization properties, conformation change, radiation-activated DNAzyme, and decoy molecules.

## 1. Introduction

Regulation of chemical or physical properties of oligodeoxynucleotides (ODNs) is important for the development of future gene diagnostics and therapy [1, 2]. Because the function of ODNs is based on their conformation and hybridization properties with their complementary DNA or RNA, various attempts have been made to manipulate these basic characteristics using chemical modification and external stimuli [3–11]. These include the binding of a metal ion to mismatched [3, 4] or modified nucleobases [5, 6], interaction of boron compounds with modified riboses [7, 8], and photochemical methods that use nitrobenzene [9, 10] or azobenzene [11] functionalities on ODNs.

High-energy ionizing radiation is an attractive stimulus for controlling the activity of biomaterials, because the radiation reaction can be controlled spatially and temporally without any additives [12, 13]. In particular, X-ray has potential because it has high live-body permeability, and thus, has been extensively used for medical treatment and diagnosis. In this paper, we describe the current state of research on controlling the function of ODNs by X-irradiation. This paper includes our recent research on the development of artificial ODNs possessing a 2-oxoalkyl group [14] or disulfide bonds [15, 16], whose properties and conformation can be regulated by X-irradiation. We applied their characteristics to regulation of hybridization, radiation-activated DNAzyme, regulation of the polymerase reaction, and conformation change of ODNs and decoy molecules for inhibition of protein-DNA interactions. 

**Figure 1:**
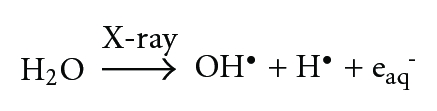
The radical products of water radiolysis.

When diluted aqueous solutions are irradiated, practically all of the absorbed energy is deposited in water molecules, and the observed chemical changes are brought about indirectly by the molecular and, in particular, the radical products of water radiolysis. It is well known that ionization and excitation of water molecules by ionizing radiation occur and generate electronically excited states (H_2_O*), radical cations (H_2_O^+•^), and dry electrons (*e*_dry_^−^). The excited water molecules H_2_O* dissociate in a homolytic manner to hydrogen atoms (H^•^) and hydroxyl radicals (OH^•^), while H_2_O^+•^ deprotonates to OH^•^, and *e*_dry_^−^ is solvated to e_aq_^−^. Eventually, e_aq_^−^, OH^•^, and H^•^ are generated as the major active species with *G* values of 280, 280, and 60 nmol/J, respectively, in the radiolysis of water (Figure 1) [17, 18]. Among the active species, we draw attention to the high reactivity of hydrated electrons and hydrogen atoms, which reduce a wide range of molecules under hypoxic conditions. We designed artificial ODNs that have high affinity for these reducing species and evaluated their reaction characteristics upon X-irradiation. 

## 2. Design and Reaction Characteristics of 2-Oxoalkyl Caged Oligodeoxynucleotides

**Figure 2:**
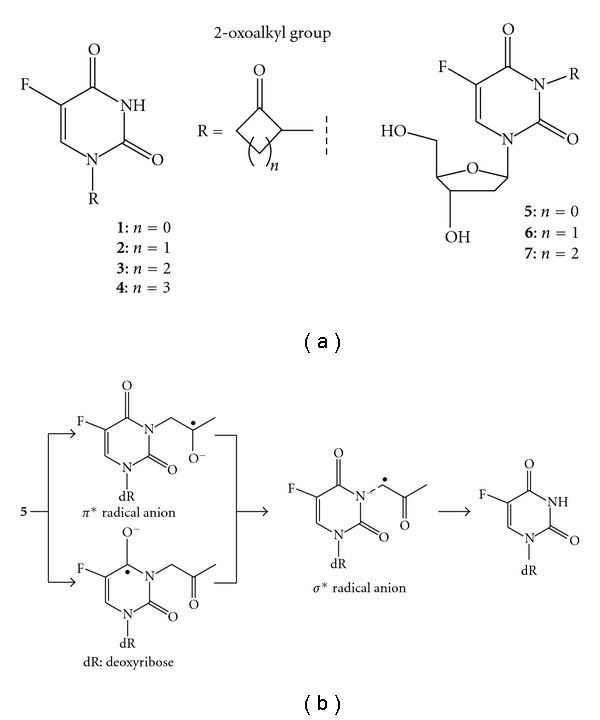
(a) Radiation-activated prodrugs of 5-flurouracil (**1–4**) and 2′-deoxy-5-fluorouridine (**5–7**) possessing 2-oxoalkyl groups. (b) Plausible mechanism for reductive release of 2-oxoalkyl group.

Previously, we designed and developed several types of radiation-activated prodrugs, which consisted of a cytotoxic agent and a substituent with high electron affinity [13, 19–22]. The nontoxic or less-toxic prodrugs underwent one-electron reduction by e_aq_^−^ to release the active agent to exhibit its inherent cytotoxicity. During the process of developing radiation-activated prodrugs, we identified a series of 2-oxoalkyl groups [19–21] that act as efficiently removable substituents by X-ray treatment under hypoxic conditions in aqueous solution (Figure 2). An activation mechanism has been proposed that the 2-oxoalkyl group undergoes one-electron reduction by e_aq_^−^ to form the corresponding π* anion radical, followed by thermal activation into the σ* anion radical, which is readily hydrolyzed to release the 2-oxoalkyl group. We have applied these characteristics of the 2-oxoalkyl group to develop several prodrugs of antitumor agents such as 5-fluorouracil and 2′-deoxy-5-fluorouridine (5-FdUrd). These contexts of previous research prompted us to apply 2-oxoalkyl substituents to radiolytic regulation of DNA duplex formation [14].

**Figure 3:**
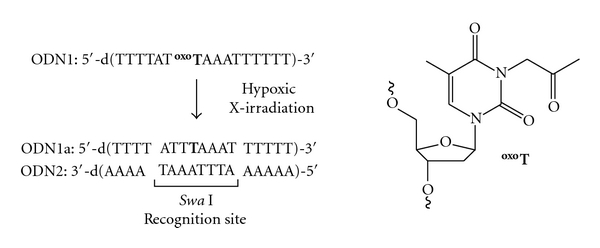
Radiolytic activation of 2-oxoalkyl caged oligodeoxynuleotides. Emergence of ordinary duplex formation by hypoxic X-irradiation.

We synthesized ODNs caged by a 2-oxopropyl group at a given thymine N(3) position (d^oxo^T), which has a characteristic structure similar to the previous radiolytic reduction-activated 5-FdUrd prodrug (Figure 3). One-electron reduction of ODNs possessing d^oxo^T, initiated by hypoxic X-irradiation, occurred to remove exclusively the 2-oxopropyl group and produce the corresponding unmodified ODNs. The unmodified 5–9 mer ODNs were formed from caged ODNs with *G* values of 100–140 nmol/J for consumption of caged ODNs and 50–110 nmol/J for formation of unmodified ODNs. On the other hand, the radiolytic removal of the 2-oxopropyl group from caged ODNs under aerobic conditions was markedly inefficient, in contrast to the prompt formation of unmodified ODNs under hypoxic conditions. Compared with the noncaged ODN 1a (*T*_*m*_ = 48.9°C), the caged ODN 1 (*T*_*m*_ = 37°C) showed a dramatic decrease in melting temperature (*T*_*m*_), indicating that the 2-oxopropyl group was effective in the destabilizing the duplex. To characterize the hybridization properties of caged ODNs, we also subjected a hypoxically irradiated ODN 1 containing d^oxo^T at the center of the *Swa* I recognition site to enzymatic digestion. After the hypoxic irradiation of ODN 1 and subsequent addition of an equimolar amount of ODN 2, which is complementary to ODN 1, treatment with *Swa* I was conducted. We observed strand cleavage of the resulting duplex at the corresponding restriction site, whereas no strand cleavage occurred when nonirradiated duplex was treated with enzyme. Thus, the ability for ordinary duplex formation was restored by hypoxic X-irradiation of caged ODNs.

**Figure 4:**
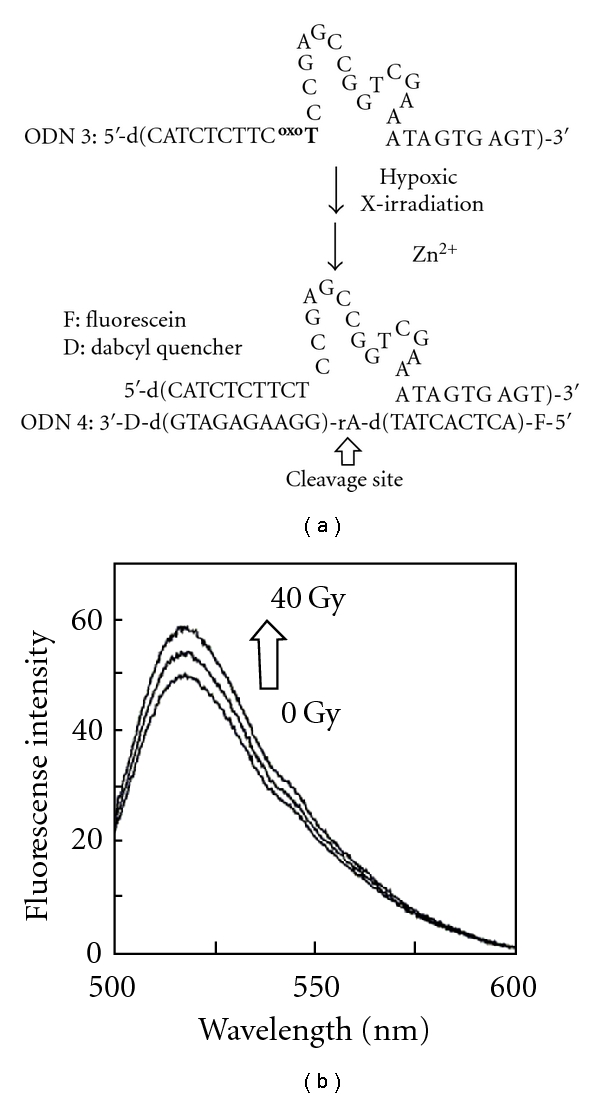
Design of caged DNAzyme activated by X-irradiation. (a) Schematic illustration of radiolytic activation of DNAzyme possessing 2-oxoalkyl group. (b) Fluorescence spectra of ODN 4 (500 nM) after addition of Zn^2+^ ion (100 *μ*M) and X-irradiated (0, 20 and 40 Gy) ODN 3 (500 nM) under hypoxic conditions.

Artificial DNA derivatives that can regulate their recognition properties and functions with the aid of external triggers are useful for a variety of applications. As an example, we tried to make DNAzyme, the function of which can be regulated by hypoxic X-irradiation. We conducted a modification of DNAzyme 17E which cleaves both RNA and DNA/RNA chimeric substrates in the presence of transition metal ions such as Zn^2+^ [23]. We designed DNAzyme 17E possessing d^oxo^T (ODN 3) at the crotch of the loop region and characterized its cleavage ability using DNA/RNA chimeric substrate (ODN 4), in which the fluorophore and quencher were incorporated into the 5′- and 3′-ends, respectively. The fluorescence emission of the fluorophore was suppressed intramolecularly by the quencher, while the cleavage of ODN 4 led to recovery of emission. Thus, we could monitor the strand cleavage of ODN 4 by fluorescence emission. Caged 33 mer DNAzyme, ODN 3, which was synthesized by automated DNA synthesis, was preirradiated and then incubated in the presence of DNA/RNA for 30 min at 25°C. As shown in Figure 4, we observed weak fluorescence emission, when ODN 3 without irradiation was incubated with ODN 4, indicating that the cleavage activity of DNAzyme 17E was efficiently reduced by incorporation of d^oxo^T into the strand. It is striking that the fluorescence intensity from ODN 4 increased with increasing radiation dose up to 40 Gy for caged DNAzyme. These results lead to the conclusion that hypoxic X-irradiation removed the 2-oxopropyl group on the caged DNAzyme, thereby resulting in the formation of active DNAzyme 17E which can cleave the DNA/RNA chimeric substrate. 

**Figure 5:**
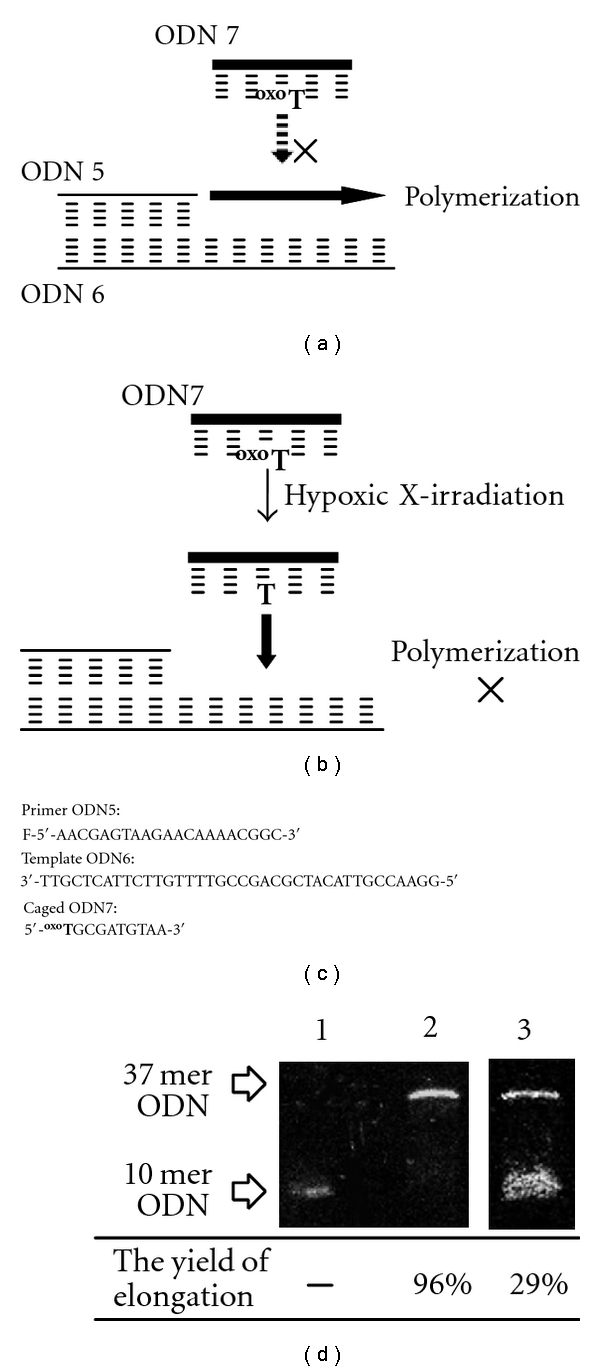
(a,b) Schematic illustration of regulation of DNA polymerase reaction by X-irradiation. The reaction in the presence of caged ODN 7 before X-irradiation (a) or after X-irradiation (b). (c) Sequences of oligodeoxynucleotides used for DNA polymerase reaction. (d) Representative gel electrophoresis of DNA elongation by the Klenow fragment of DNA polymerase I. ODN 7 (100 *μ*M) was X-irradiated (1200 Gy) under hypoxic conditions, and then ODN 6 (5 *μ*M), fluorescein-labeled ODN 5 (5 *μ*M), and enzyme (0.2 unit) were added. After the incubation for 15 min at 32°C, the elongation was monitored by gel electrophoresis. Lane 1: marker (20 mer), Lane 2: the ODN 7 was added before X-irradiation. Lane 3: the ODN 7 was added after X-irradiation (1200 Gy).

We also applied a caged ODN to regulation of the DNA polymerase reaction [24]. DNA elongation of primer ODN 5 by the Klenow fragment of DNA polymerase I was regulated by caged ODN 7.  Figure 5 shows the protocol and a representative gel picture. When 10 mer caged ODN 7 without X-irradiation was added to the mixture of primer ODN 5, template ODN 6 and enzyme, DNA polymerization occurred to form a 37 mer ODN, which was complementary to ODN 6 with a yield of 96%. This result indicates that caged ODN 7 did not act as a suppressor of enzymatic reaction as ODN 7 could not hybridize with template ODN 6 because of steric hindrance of the 2-oxoalkyl group. In contrast, X-irradiation resulted in removal of the 2-oxoalkyl group from ODN 7, and thereby addition of X-irradiated ODN 7 prevented DNA elongation by duplex formation with template ODN 6. The yield for elongation decreased to 29%. Thus, DNA polymerization has been successfully regulated by X-irradiation. 

## 3. Radiolytic Reduction of Oligodeoxynucleotides Possessing Disulfide Bonds

Disulfides, thiols, and their corresponding radicals have been shown to induce unique reactions, including formation or rupture of covalent bonding under reduction conditions. For example, the disulfide radical anion (RSSR^-•^) generated by one-electron reduction of a disulfide is converted into a sulfide anion (RS^−^) and a thiyl radical (RS^•^) [25, 26]. Sulfide anions tend to undergo disulfide exchange reactions in the presence of disulfide compounds, while thiyl radicals abstract a hydrogen atom to form thiols [27–29]. In addition, hydrogen atoms are assumed to reduce disulfide to form a radical anion [16]. These reaction characteristics motivated us to investigate radiolytic reduction of ODNs possessing disulfide bonds [15, 16].

**Figure 6:**
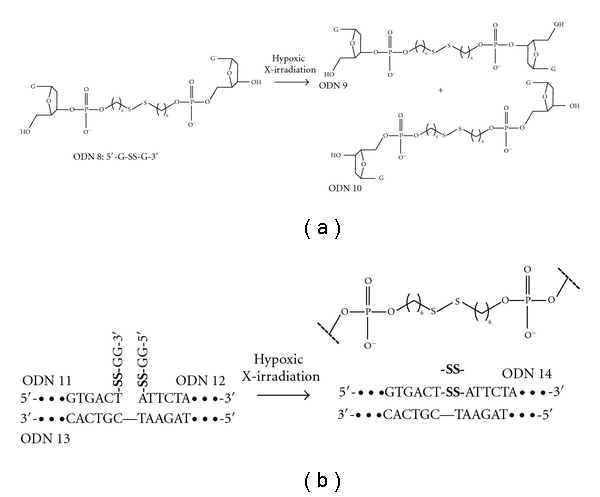
(a) Radiolytic strand exchange reaction of dinucleotide unit possessing disulfide bond. (b) Radiolytic template-directed ligation of oligodeoxynucleotides possessing disulfide bond.

We initially conducted radiolytic reduction of dinucleotides possessing a disulfide bond (ODN 8) [15]. After the hypoxic irradiation, two ODNs (ODN 9 and ODN 10) were formed as reaction products via an intramolecular strand-crossing reaction concomitantly with the regeneration of ODN 8. Mechanistic studies revealed that this reaction proceeded with multiple turnover process. We subsequently applied these unique reaction characteristics to template-directed strand crossing. In the presence of a complementary ODN (ODN 13), two ODNs possessing a disulfide bond (ODN 11 and ODN 12) produced a specified ODN (ODN 14) via interstrand crossing upon hypoxic irradiation. Radiolytic reduction of ODNs possessing a disulfide bond resulted in the easy preparation of a specified ODN (Figure 6).

**Figure 7:**
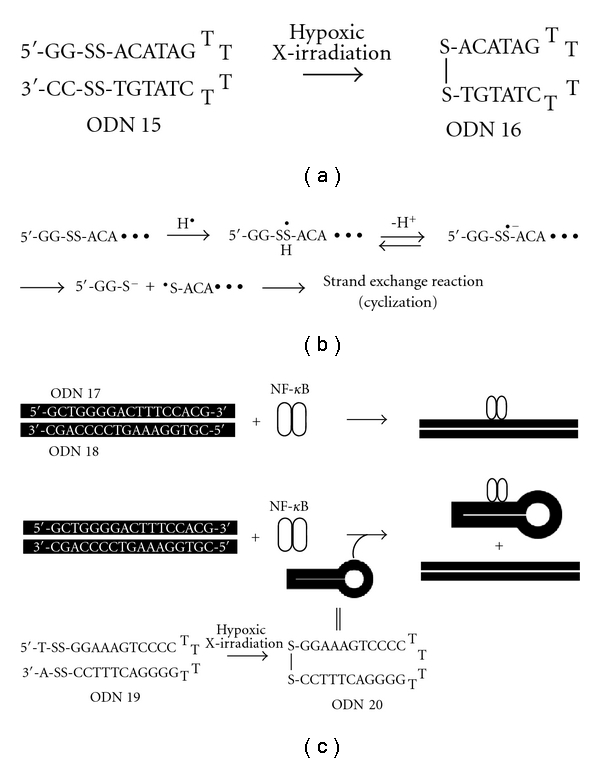
(a) Radiolytic cyclization of hairpin-type oligodeoxynucleotides possessing disulfide bonds. (b) The possible reaction mechanism for the cyclization of ODN 15 upon X-ray irradiation. (c) Cyclized oligodeoxynucleotides used for the transcriptional decoy strategy.

As well as these unique reaction characteristics, the disulfide bond plays an important role in the construction of the secondary or tertiary structure of DNA architecture [30–32]. Disulfide bond formation and dissociation are significantly affected by the redox state, and therefore the higher-order structure can be controlled by redox reactions. We next conducted the radiolytic reduction of hairpin-type ODNs possessing two disulfide bonds in order to establish guides for the design of radiation-associated systems to regulate the DNA higher-order structure. We carried out the radiolytic reduction of 20 mer hairpin-type ODN 15 by the X-radiolysis of an argon-purged aqueous solution in the usual manner. As shown in Figure 7, the corresponding cyclized ODNs, which were identified by HPLC analysis and ESI-TOF mass measurements, were formed with *G* values of 100 nmol/J for the formation of cyclized ODNs. We also confirmed that the reaction efficiency decreased substantially upon increasing the ODN chain length, whereas it was improved by elevating the reaction temperature. 

In a separate experiment, we characterized the effect of reactive species generated by radiolysis of water on the cyclization of ODNs possessing disulfide bonds. When hairpin-type ODN 15 was X-irradiated in an aqueous solution containing OH^•^-scavenging 2-methyl-2-propanol purged with nitrous oxide (N_2_O) gas [17], which efficiently captures e_aq_^−^, cyclization was moderately suppressed. On the other hand, the formation of cyclized ODNs was dramatically suppressed in the presence of 2-propanol, which captures both OH^•^ and H^•^. These results strongly underline the importance of H^•^ as well as e_aq_^−^ for the activation and strand-crossing reaction of disulfide bonds. 

End-capped DNA has recently attracted much attention in the field of gene therapy because of its high stability under biological conditions [33–37]. Further attempts have been made to apply radiolytically cyclized ODN to a decoy strategy directly targeting transcription factors [16]. We prepared a 28 mer hairpin-type ODN 19 possessing two disulfide bonds and characterized its radiolytic reaction, stability, and binding properties against NF-*κ*B. Similar to the results described above, ODN 19 was efficiently cyclized by hypoxic X-irradiation. The cyclized ODN 20 was almost-totally resistant to enzymatic digestion by snake venom phosphodiesterase, while prompt digestion was observed for hairpin-type ODN 19. We next demonstrated that radiolytically cyclized ODN 20 interacts with NF-*κ*B as a decoy by in vitro competition assay. An electrophoretic mobility shift assay revealed that addition of cyclized ODN 20 effectively prevented the binding of NF-*κ*B with a control duplex (ODN 17/ODN 18) in a sequence-selective manner. Thus, the radiolytic formation of cyclized ODNs is promising for a decoy strategy that controls gene expression by inhibition of specific transcription-regulation proteins. 

## 4. Conclusion

Artificial oligonucleotides whose hybridization properties, conformation, and functions can be regulated by external stimuli are applicable to a wide range of gene research areas. From this perspective, we have summarized our recent advances in the development of caged oligonucleotides activated by X-irradiation. Radiolytic reduction by reactive species such as hydrated electrons or hydrogen atoms caused removal of the 2-oxoalkyl group on base units and exchange reaction of the disulfide bond. The hybridization properties and conformation of ODNs possessing these substituents are modulated by X-irradiation, and thereby they show promise as radiation-activated DNAzyme as modulators of the DNA polymerase reaction and as decoy molecules. However, these artificial ODNs are premature for practical use because high radiation doses are required to activate the caged ODNs. Because high doses of X-irradiation may cause damage to cellular DNA, it is difficult to apply the present system to in vivo experiments as it stands. One of the key strategies to overcome this problem is further chemical modification of radiation-activated substituents that have a high affinity for hydrated electrons and hydrogen atoms. Our current interest focuses on the construction of improved caged DNA systems that are highly sensitive to small doses of X-irradiation and their application to in vivo gene regulation.
